# Relapse of chronic melioidosis in a paediatric cystic fibrosis patient: first case report from Malaysia

**DOI:** 10.1186/s12879-018-3371-7

**Published:** 2018-09-05

**Authors:** Vanitha Mariappan, Surendran Thavagnanam, Kumutha Malar Vellasamy, Cindy Ju Shuan Teh, Nadia Atiya, Sasheela Ponnampalavanar, Jamuna Vadivelu

**Affiliations:** 1 Department of Medical Microbiology Faculty of Medicine, University of Malaya, 50603 Kuala Lumpur, Malaysia; 2 University Malaya Pediatric and Child Health Research Group, Faculty of Medicine, University of Malaya, 50603 Kuala Lumpur, Malaysia; 3 Department of Medicine, Faculty of Medicine, University of Malaya, 50603 Kuala Lumpur, Malaysia

## Abstract

**Background:**

*Burkholderia pseudomallei* is the causative agent of melioidosis, which is a potentially life threatening disease endemic in Southeast Asian countries. In Malaysia, cystic fibrosis (CF) is an uncommon condition. The association between CF and *B.pseudomallei* infections has been reported previously. However, this is the first case report of a pediatric melioidosis relapse and co-infection with other Gram-negative bacteria in Malaysia.

**Case presentation:**

A 14-year-old Chinese Malaysian boy presented with a history of recurrent pneumonia, poor growth and steatorrhoea since childhood, and was diagnosed with CF. *B. pseudomallei* was cultured from his sputum during three different admissions between 2013 and 2016. However, the patient succumbed to end stage of respiratory failure in 2017 despite antibiotics treatment against *B.pseudomallei*. The isolates were compared using multilocus-sequence typing and repetitive-element polymerase chain reaction (PCR), and confirmed that two of the isolates were of same sequence type, which may indicate relapse.

**Conclusions:**

CF patients should be aware of melioidosis in endemic regions, as it is an emerging infectious disease, especially when persistent or recurrent respiratory symptoms and signs of infection occur. The high prevalence rates of melioidosis in Malaysia warrants better management options to improve quality of life, and life expectancy in patients with CF. Travel activities to endemic regions should also be given more consideration, as this would be crucial to identify and initiate appropriate empiric treatment.

## Background

Cystic fibrosis (CF) is the most common genetic disorder among Caucasians, associated with bacterial lung infection. Authors have reported on *Burkholderia pseudomallei* infection among the CF patients [1]. It is however notable that CF is a rare disease in Malaysia. Melioidosis is a potentially fatal infection that is endemic in the tropical and subtropical regions especially in Southeast Asia and Northern Australia [2]. *B.pseudomallei*, the causative agent of melioidosis, is commonly found in soil and contaminated water and the mode of transmission can be through inhalation, ingestion, inoculation or direct entry into the blood stream via wounds or skin abrasions [3]. It is known to remain persistent in the host for many years after initial infection despite antibiotic treatment [4]. Due to the severity of the disease, aerosol infectivity and intrinsic resistance to a large number of antimicrobial agents, *B.pseudomallei* has been classified as a potential agent for bioterrorism and has been indicated as an emerging infectious disease [5]. Melioidosis can clinically manifest as chronic pneumonia, asymptomatic infection, multiple internal organ abscesses and septic shock [6]. Melioidosis in adults are more widely reported as compared to the pediatric population (5–15%) [7, 8]. Here, we report a relapse case of chronic pulmonary meliodosis in a pediatric patient with CF who travelled to *B.psuedomallei* endemic area. Our aim is to create awareness among physicians and CF patients regarding endemic regions for melioidosis and possible infections or relapse.

## Case presentation

A 14-year-old Chinese Malaysian boy presented to University Malaya Medical Centre, Kuala Lumpur in September 2013 with history of recurrent pneumonia, poor growth and steatorrhoea since childhood. He had finger clubbing and bronchiectasis. Later, he was diagnosed with CF and *Pseudomonas aeruginosa* was isolated from his sputum. He received 3 weeks of intravenous ceftazidime (50 mg/kg/dose, QDS) and gentamicin (5 mg/kg/dose, OD). He was discharged with azithromycin (5 mg/kg EOD), nebulised gentamicin (80 mg BD) amongst other CF-related medications. In November 2013, he was readmitted with a pulmonary exacerbation and his sputum sample grew methicillin-resistant *Staphylococcus aureus* (MRSA) and he received intravenous vancomycin, oral rifampicin (300 mg BD) and sodium fusidate (500 mg TDS) with significant clinical improvement. During a follow-up visit in December 2013, he had a productive cough but was apyrexic. He was empirically treated with oral ciprofloxacin (750 mg BD) and the sputum sample later isolated *B.pseudomallei.* As he clinically improved*,* the treatment regimen remained unchanged. Subsequently, the repeat sputum samples were negative for *B.pseudomallei* and he continued to remain active with good exercise tolerance and relatively stable lung function. It is noteworthy that he had been residing in an urban area of non-endemicity for melioidosis and there were no other known risk factors identified.

In August 2014, he was admitted with another pulmonary exacerbation and his cultured sputum grew *B.pseudomallei* and *Pseudomonas* spp. His chest radiograph showed diffused interstitial changes with bronchiectasis throughout both lungs with minimal pleural effusions. He received 2 weeks of intravenous ceftazidime (2 g; 6hourly) and amikacin (720 mg; 15 mg/kg/OD). Upon completion of antibiotics, he remained afebrile and the chest auscultatory findings improved. He was discharged with 6 months of oral doxycycline, and co-trimoxazole to treat the *B.pseudomallei* and 3 months of nebulized amikacin for chronic *P.aeruginosa*. He still continued on his alternate day of azithromycin (250 mg).

A detailed travel history revealed that in June 2014, he visited recreational parks in Sabah, Malaysia. During the visit, he went jungle trekking, snorkeling and dipped in a hot-water spring. It is noteworthy that melioidosis is endemic in Sabah, one of the two East Malaysian states on the island of Borneo, where *B.pseudomallei* prominently occurs in soil and water. Later then, he was admitted 3 monthly for antibiotic tuning and his sputum culture had no specific bacterial growth.

In August 2015, there was a decline in his lung function tests with deterioriorating cough. A bronchoscopy was performed and *Burkholderia cepacia* was isolated from his bronchoalveoloar lavage specimen, while acid-fast bacillus (AFB) smear was weakly positive. Initially, the patient was treated with intravenous imipenem and ceftazidime for 3 weeks but had recurrence of fever. However, the sputum AFB smears remained positive although the suspected nontuberculous mycobacterium could not be isolated despite various culturing techniques. Therefore, the antibiotics were changed to intravenous meropenem, doxycycline, amikacin and oral clarithromycin to treat both the *B.cepacia* and the suspected nontuberculous mycobacterium.

**Fig. 1:**
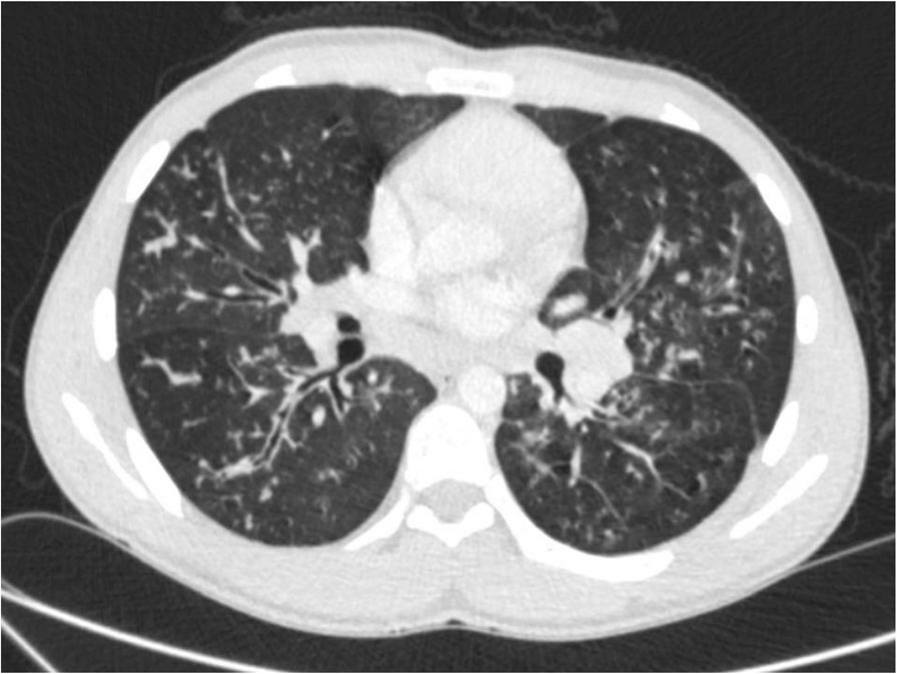
Computed tomography of chest showing bronchiectasis changes and peribronchovascular thickening seen throughout both lung fields. Tree in bud appears in the periphery predominantly in the right middle and lower lobe and lingular segment of the left upper lobe

Upon discharge, he had been continuing with amoxicillin-clavulanate and doxycycline for 6 months, which helped with weight gain and secretion reduction. Repeat AFB smear remained negative for the subsequent 5 months. However, in January 2016, further decline in his lung function was observed with worsening respiratory symptoms. A chest computed tomography showed worsening bronchiectasis, tree in bud appearance in the lung peripheries, patchy consolidation and several enlarged lymph nodes at the right paratracheal region (Fig. 1). His sputum sample grew *P.aeruginosa* and was also strongly positive for AFB. Intravenous meropenem and ceftazidime (for *P.aeruginosa*) and combination therapy of rifampicin, ethambutol, azithromycin and nebulized amikacin (for nontuberculous mycobacterium) was started. He improved clinically and was discharged with the above oral medications for 6 months.

*B.pseudomallei* was isolated once again in April 2016 and he was treated with intravenous amoxicillin-clavulanate and ceftazidime for 3 weeks. He was then discharged with 6 months of oral amoxicillin-clavulanate. However, in May 2016, his antibiotics were changed to levofloxacin (750 mg) and clarithromycin (500 mg). Following that, for the next 8 months, his sputum sample continued to grow *B.pseudomallei* but was negative for AFB. Despite many admissions for intravenous antibiotics against *B.pseudomallei*, the patient passed away from end stage respiratory failure in February 2017. Bacteriological reports were reviewed, and over the 3 years, the patient had several infective exacerbations and his sputum samples grew Gram-negative organisms that were later identified to be *B.pseudomallei*, *Pseudomonas* spp., *P.aeruginosa*, or *B.cepacia* (Table 1).

**Table 1:** Documented exacerbations with Gram-negative organisms reported to be *Burkholderia pseudomallei*, *Pseudomonas sp*., *Pseudomonas aeruginosa*, or *Burkholderia cepacia* (December 2013 – February 2017)

Organism	Source / Date	Antibiotic susceptibility test			Other data
		Resistant	Intermediate	Sensitive	
*Burkholderia pseudomallei*	Sputum (December 2013)	• Amoxicilin-clavulanate		• Ceftazidime • Doxycycline • Imipenem • Co-timoxazole	*Pus cell: > 25/lpf *Epithelial cell: 3/lpf
*Burkholderia pseudomallei*	Sputum (August 2014)			• Amoxicilin-clavulanate • Ceftazidime • Doxycycline • Imipenem • Co-timoxazole	*Pus cell: > 25/lpf *Epithelial cell: 5/lpf
*Pseudomonas*	Sputum (August 2014)	• Gentamicin		• Ceftazidime • Piperacillin-tazobactam	*Pus cell: > 25/lpf *Epithelial cell: 15/lpf
*Burkholderia cepacia*	Bronchoalveolar lavage (August 2015)	• Amoxicilin-clavulanate • Amikacin • Azithromycin • Ciprofloxacin • Gentamicin • Imipenem • Piperacillin-tazobactam		• Co-trimoxazole • Meropenem • Ceftazidime	*No virus detected *AFB weakly positive *No Mycobacterium
*Burkholderia cepacia*	Sputum (November 2015)	• Imipenem • Co-trimoxazole		• Meropenem • Ceftazidime	*Pus cell: 0/lpf *Epithelial cell: 1/lpf *AFB negative
*Pseudomonas aeruginosa*	Sputum (January 2016)	• Azithromycin		• Amikacin • Ceftazidime • Ciprofloxacin • Gentamicin • Meropenem	–
*Burkholderia pseudomallei*	Sputum (March 2016)	• Co-trimoxazole			*Pus cell: 15/lpf *Epithelial cell: 0/lpf *AFB positive
*Burkholderia pseudomallei*	Sputum (April 2016)	• Co-trimoxazole			*AFB positive
*Burkholderia pseudomallei*	Sputum (May 2016)		• Doxycycline	• Ceftazidime • Imipenem Amoxicilin-clavulanate	*AFB positive *Acute duodenitis *No malignancy *Neutrophilic infiltration *OGDS - Pangastritis *Rapid urease test negative
*Burkholderia pseudomallei*	Sputum (May 2016)	• Co-trimoxazole		• Amoxicillin-clavulanate	
*Burkholderia pseudomallei*	Sputum (May 2016)			• Sensitive to all antibiotics	
*Burkholderia pseudomallei*	Sputum (June 2016)			• Amoxicillin-clavulanate • Doxycycline	*AFB positive
*Burkholderia cepacia*	Sputum (December 2016)	• Ciprofloxacin • Imipenem • Co-trimoxazole		• Ceftazidime • Meropenem	
*Burkholderia pseudomallei*	Bronchoalveolar lavage (February 2017)				*Candida parapsilosis (10^4^–10^5^) *AFB negative
*Burkholderia pseudomallei*	Sputum (February 2017)	• Doxyccyline • Co-trimoxazole		• Amoxi-clavunate • Ceftazidime • Imipenem	

All the isolates were found to have different susceptibility patterns (resistant to co-trimoxazole; intermediate to doxycycline and susceptible to all other antibiotics). The *B.pseudomallei* isolated in 2013, 2014 and 2016 (UMC083, UMC 082 and UMC114, respectively), were also further confirmed as *B.pseudomallei* using API 20NE (Biomerieux, France), Ashdown agar and also an in-house polymerase chain reaction (PCR) using specific primers [9]. However, we were not able to obtain the *B.pseudomallei* isolated in 2017.

The isolates were characterized by multilocus sequence typing (MLST), a method of molecular subtyping that compares sequences of seven housekeeping genes [10], and repetitive-element PCR (rep-PCR). It appeared that these isolates were of two different sequence type (ST); ST51 (UMC083 and UMC114), which is a common ST found widely in Malaysia, Thailand, Singapore, Hong Kong and China, and ST1644, (UMC082) a new ST. The STs were deposited in the *B.pseudomallei* MLST database (https://pubmlst.org/bpseudomallei/).

## Discussion and conclusions

Colonisation and infection with *B.cepacia* complex is a well-known issue in CF patients and associated with declining lung function [11]. Similarly, speculation as to whether alterations in CF lungs predispose individuals to infection with *B.pseudomallei* has always existed. Over time, many researchers have reported the association between CF patients and *B.pseudomallei* infections, especially those who traveled to the melioidosis endemic regions [12, 13]. However, not many groups have described melioidosis relapse cases among the CF patients. It is noteworthy to mention that to date, no vaccine is available to prevent the disease [14]. Asiah et al. [15] was the first to report case of a CF pediatric patient in Malaysia.

In our case, the patient was having relapse of *B.pseudomallei* infections and we postulate that UMC114 may be a relapse strain of UMC083, despite 120 weeks of treatment. However, the UMC082 was a new infection, which was probably acquired during his vacation in Sabah. Cases have been reported in adults from Sabah, and a sero-prevalence study of military personnel in Sabah found that almost 60% had antibodies to *B.pseudomallei* [16]. Or it could also be possible that the patient was infected with two (or possibly more) strains during the original infection event, but due to minimal sampling and culturing efforts, this mixture was not detected until ST1644 was identified one year later. We can also postulate that the patient acquired a new infection with ST1644 from his hometown, but this clone has not been previously identified in Malaysia. We cannot completely rule out this possibility of a primary infection from his area of residence, as prevalence of *B.pseudomallei* in the Malaysian environment is still quite poorly understood. However, it is also likely that there are many undiscovered STs in the Malaysian environment.

In general, the diagnosis of pulmonary melioidosis in patients with CF could be complicated for several reasons including over growth of other organisms, presence of non-culturable *B.pseudomallei* and cross-reaction with other closely related organisms. As for better management options, quality of life and life expectancy, melioidosis should be considered as a differential diagnosis in patients with CF presenting with chronic pneumonia, especially in those residing or have travelled to a melioidosis endemic area, although it is not possible to quantify the level of risk for individual travelers. If respiratory health of the CF patient deteriorates following a trip to these areas, the patient should immediately consult the CF team and ensure to give full details of clinical and travel history. At the same time, the physicians should also be keen in taking detail travel history. This is crucial to identify and initiate appropriate empiric treatment.

We suggest that the CF pediatric patients in endemic regions should reduce activities that may have close contact with soil or water as it may aid in decreasing the chances of *B.pseudomallei* infections. They should also be aware of *B. pseudomallei* infections especially when persistent or recurrent respiratory symptoms and signs of infection occur and there is an unexpected poor response to standard antibiotic therapy. In view of the chronicity of the underlying disease, further understanding of the virulence factors of infective organisms, may help to decide on the optimal treatment regimen.

## Abbreviations

AFB: Acid-fast bacillus
BD: Bis in die – twice a day
CF: Cystic fibrosis
MLST: Multilocus sequence typing
MRSA: Methicillin-resistant *Staphylococcus aureus*
OD: Omne in die – once a day
PCR: Polymerase chain reaction
QDS: Quater die sumendum - 4 times a day
rep-PCR: Repetitive-element PCR
ST: Sequence type
TDS: Ter die sumendum – 3 times a day
